# Reasons for Ineligibility in Phase 1 and 2A HIV Vaccine Clinical Trials at Kenya Aids Vaccine Initiative (KAVI), Kenya

**DOI:** 10.1371/journal.pone.0014580

**Published:** 2011-01-21

**Authors:** Gloria S. Omosa-Manyonyi, Walter Jaoko, Omu Anzala, Hilda Ogutu, Sabina Wakasiaka, Roselyn Malogo, Jacqueline Nyange, Pamela Njuguna, Jeckoniah Ndinya-Achola, Kirana Bhatt, Bashir Farah, Micah Oyaro, Claudia Schmidt, Frances Priddy, Patricia Fast

**Affiliations:** 1 Kenya AIDS Vaccine Initiative (KAVI), University of Nairobi, Nairobi, Kenya; 2 International AIDS Vaccine Initiative (IAVI), New York, New York, United States of America; University of Cape Town, South Africa

## Abstract

**Background:**

With the persistent challenges towards controlling the HIV epidemic, there is an ongoing need for research into HIV vaccines and drugs. Sub-Saharan African countries - worst affected by the HIV pandemic - have participated in the conduct of clinical trials for HIV vaccines. In Kenya, the Kenya AIDS Vaccine Initiative (KAVI) at the University of Nairobi has conducted HIV vaccine clinical trials since 2001.

**Methodology:**

Participants were recruited after an extensive informed consent process followed by screening to determine eligibility. Screening included an assessment of risk behavior, medical history and physical examination, and if clinically healthy, laboratory testing. In the absence of locally derived laboratory reference ranges, the ranges used in these trials were derived from populations in the West.

**Principal findings:**

Two hundred eighty-one participants were screened between 2003 and 2006 for two clinical trials. Of these, 167 (59.4%) met the inclusion/exclusion criteria. Overall, laboratory abnormalities based on the non-indigenous laboratory references used were the most frequent reasons (61.4%) for ineligibility. Medical abnormalities contributed 30.7% of the total reasons for ineligibility. Based on the laboratory reference intervals now developed from East and Southern Africa, those ineligible due to laboratory abnormalities would have been 46.3%. Of the eligible participants, 18.6% declined enrolment.

**Conclusions:**

Participant recruitment for HIV vaccine clinical trials is a rigorous and time-consuming exercise. Over 61% of the screening exclusions in clinically healthy people were due to laboratory abnormalities. It is essential that laboratory reference ranges generated from local populations for laboratory values be used in the conduct of clinical trials to avoid unnecessary exclusion of willing participants and to avoid over-reporting of adverse events for enrolled participants.

**Trial registration:**

Protocol IAVI VRC V001 [1]. ClinicalTrials.gov NCT00124007 Protocol IAVI 010 [2] *(registration with ClincalTrials.gov is in progress)*

Protocols IAVI 002 and IAVI 004 are Phase 1 trials only mentioned in introductory paragraphs; details will not be reported. Registration was not required when they were conducted.

## Introduction

The majority of the people living with HIV are in sub-Saharan Africa (68%) with some countries having disturbingly high HIV prevalence rates. In 2009, Sub-Saharan Africa had an overall HIV prevalence rate of 5% [3]. In some populations, the HIV prevalence rate is much higher than average; for example in 2009 the prevalence rates for Botswana were 24.8%, for South Africa 17.8%, and for Swaziland 25.9% [3]. Although the current control measures may have lowered the HIV prevalence in some countries, scientists generally believe that a safe, effective, accessible and affordable preventive HIV vaccine is the best hope for stopping the HIV epidemic. In this regard, sub-Saharan Africa, which endures most of the HIV epidemic, must play its rightful role in research and development, not only of preventive HIV vaccines, but also discovery and development of new anti-retroviral drugs.

Several African countries have participated in HIV vaccine research. Since 2001, the Kenya AIDS Vaccine Initiative (KAVI) at University of Nairobi (Kenya) has conducted three phase 1 and one phase 2A HIV vaccine clinical trials.

The phase 1 trials tested the following:

- an HIV clade A plasmid DNA (pTHr.DNA.HIVA) vaccine - Protocol IAVI 002 (2001) [4], [5], [6]
- a recombinant vaccine vectored by modified vaccinia virus Ankara (MVA); MVA.HIVA - Protocol IAVI 004 (2002) [4], [5], [6]
- the VRC recombinant replication-defective Adenovirus subtype 5-vectored multi-clade vaccine (Vaccine Research Center) NIH, VRC HIV-1 rAd5), either alone or as boost following VRC HIV-1 DNA vaccine - Protocol IAVI V001 (2006) [1]

The phase 2A trial tested the following:

- a plasmid DNA.HIVA as prime followed by MVA.HIVA as boost – Protocol IAVI 010 (2003). [2], [5], [6]

To determine eligibility, potential participants in all these trials went through a rigorous screening process, and were only enrolled after meeting inclusion/exclusion criteria specific to the respective study protocol. Generally, these requirements included healthy, HIV negative, 18 to 50 year-old subjects, who had not participated in any trial of an investigational product, and were willing and able to give informed consent. In addition to an HIV risk assessment, participants had clinical screening that included medical history, medical examination and laboratory testing. Study specific requirements included chest x-ray to exclude active tuberculosis and stool microscopy for intestinal parasites in two early studies conducted at KAVI [4], [5]. These screening tests excluded few subjects.

This paper examines the reasons potential participants were ineligible for enrollment in two vaccine clinical trials conducted at KAVI during the period 2003 to 2006, ie, Protocols 010 and V001.

## Materials and Methods

### Ethics Statement

Scientific and ethical clearance to conduct these studies, including the informed consent document, was obtained from Kenyatta National Hospital/University of Nairobi Ethics and Research Committee (KNH/UONERC). In addition, the V001 study protocol was reviewed and approved by the Pharmacy and Poisons Board. As per the Kenyan requirements, the National Council for Science and Technology received the protocols for information.

### Participant recruitment methods

The participant/volunteer recruitment involved the use of a peer-leader approach, as well as electronic and print media. The peer-leaders were members chosen according to the communities they represented and were used to gain access to communities for recruitment seminars. Information posters on the studies were placed in public places, such as shopping centers and bus stations. The informed consent process started with peer-leader-organized informational seminars held in various communities, at institutions of higher learning, such as polytechnics, medical training colleges, and universities, and other organizations based in Nairobi. At these seminars, those interested in participating in the clinical vaccine trials were invited to a recruitment seminar at the study site at KAVI. Those who attended the recruitment seminars were then invited to individualized sessions with the study nurse-counselors or physicians where detailed information on participation in the clinical trials was given, including discussion of the informed consent document. During the sessions, individuals' questions were answered comprehensively, and the participants were provided the informed consent document to take home for review. In addition, individuals were counseled on contraception and low-risk behavior for HIV infection. These individualized information sessions improved potential participants' understanding of the study's requirements. Some of them, realizing they did not qualify for the study, excluded themselves and thus did not proceed further. Interested individuals were then scheduled for a screening visit to assess their eligibility. On average, each participant attended a recruitment seminar and three individualized information sessions prior to the screening visit; this process required about two weeks.

### Screening procedures

A study nurse-counselor verified each participant's identity and age, reviewed the informed consent document with participants, and then assessed their understanding of the trial using a standard assessment of understanding questionnaire. Only those who passed this assessment were allowed to participate in the trials and were asked to provide a signed informed consent. Thereafter, a screening questionnaire was administered and HIV risk assessment performed. For participants who were determined to practice low-risk behavior for HIV infection, the study physician obtained medical history and performed a complete physical examination; vital signs; height and weight; examination of skin, respiratory, central nervous, cardiovascular, and abdominal systems; and an assessment of cervical and axillary lymph nodes. Laboratory tests were not performed on participants with any clinically significant abnormality as determined from medical history or physical examination. Individuals were excluded from the study if they had history of immunodeficiency or autoimmune disease; or had used systemic corticosteroids, immunosuppressive, antiviral, anticancer, or other medications considered significant by the investigator within the previous 6 months. They were also excluded if they had any clinically significant acute or chronic medical condition that was considered progressive, or in the opinion of the investigator, would make the participant unsuitable for the study.

Participants who had normal findings from their medical history and physical examination received pre-HIV-test counseling before blood (venous) and urine specimen collection. Specimen collection occurred between 0800 and 1400 hours. Hematology and clinical chemistry assays were completed on the day of sample collection; the other tests were performed within one week. All the assays were performed at the KAVI Laboratory, Department of Medical Microbiology in the University of Nairobi. This laboratory had internal quality assurance systems in place and received international Good Clinical and Laboratory Practice (GCLP) accreditation in the year 2005. Laboratory personnel compiled results that were reviewed by the laboratory manager and then dispatched to the clinic within five days of sample collection. In the clinic, the study physicians and nurses reviewed each participant's screening results to determine eligibility for enrolment. For the hematological and clinical chemistry parameters, eligibility was determined using a set of modified reference ranges derived from populations in the US (Table 1) [7].

**Table 1:** Comparison/consensus intervals for select hematology and biochemistry assays for US, Africa, and Protocols IAVI 010 and V001.

Analytes	East/South Africa Consensus Interval+	US-Based Comparison Interval*	Modified US Comparison Interval**	Units
**Chemistry**				
Creatinine	47–109	0–133	55–133	µmol/L
AST (SGOT)	14–60	0–35	<40	IU/L
ALT (SGPT)	8–61	0–35	<40	IU/L
Bilirubin, direct	0.4–4.8	1.7–5.1	<7	µmol/L
Bilirubin, total	2.9–37.0	5.1–17	<17	µmol/L
**Hematology**				
RBC: male & female	3.8–6.4	4.0–5.9	4.1–6.1	×10^6^ cells/µL
Hemoglobin: male	12.2–17.7	13.5–17.5	12–18	g/dL
Hemoglobin: female	9.5–15.8	12.0–16.0	12–18	g/dL
Hematocrit: male	35.0–50.8	41–53	34–52	%
Hematocrit: female	29.4–45.4	36–46	34–52	%
Platelets	126–438	150–350	130–550	×10^3^ cells/µL
Total WBC	3.1–9.1	4.5–11.0	3.3–11.0	×10^3^ cells/µL
Neutrophil count	1.0–5.3	1.8–7.7	1.5–8.0	×10^3^ cells/µL
Neutrophil (%)	25–66	40–70	45–75	%
Lymphocyte count	1.2–3.7	1.0–4.8	0.8–4.9	×10^3^ cells/µL
Lymphocyte (%)	23–59	22–44	16–50	%
Monocyte (%)	4.5–13.1	4–11	4–11	%
Eosinophil (%)	0.8–21.8	0–8	0–8	%
CD4 count	457–1628	518–1981	518–1981	Cells/µL
CD8 count	230–1178	270–1335	270–1335	cells/µL

+ From Karita E, et al [8].

*From Kratz A, et al [7].

**Comparison intervals for Protocols 010 and V001.

The hematology assays performed during screening included white blood cell counts, red blood cell counts, differential, and hemoglobin. Clinical chemistry assays included direct and total serum bilirubin, alanine aminotransferase (ALT/SGPT), aspartate aminotransferase (AST/SGOT), alkaline phosphatase, and serum creatinine. Other tests included urinalysis, CD4 and CD8 cell counts, HIV ELISA, Hepatitis B surface antigen, Hepatitis C antibodies, syphilis serology, urine pregnancy, and antinuclear Anti-ds DNA antibodies (ANA). In Protocol V001, laboratory parameters did not include eosinophil counts, total or direct serum bilirubin, alkaline phosphatase, AST (SGOT), or Anti-ds DNA.

### Instrumentation and assays employed in the laboratory assays

- Vitalab Selectra E Chemistry Analyzer: Vital Scientific, Netherlands. Spectrophotometric, 32 analyte, open-system analyzer.
- COULTER® Ac·T™ 5diff OV: Beckman Coulter, Inc. Brea, California, USA. 5-part differential analysis system employs absorbance cytochemistry and Volume (AcV) technology.
- Vironostika® HIV-1 Antigen: Biomerieux, Inc, Durham, NC. Microelisa test for the detection of p24 core HIV-1 antigen in serum, plasma or culture supernatant (used in Protocol 010 only).
- Vironostika® HIV Uni-Form II Ag/Ab: Biomerieux, Inc, Durham, NC. One step Microelisa test for the immunological detection of HIV-1, HIV-1 Group O and HIV-2 infection (used in Protocol V001 only).
- Detect HIV I/II ™: Adaltis, Rome, Italy.
- Monolisa™ HBs Ag ULTRA Assay: Bio-Rad Laboratories, Inc, Hercules, California, USA. A one-step enzyme immunoassay for the detection of the surface antigen of the Hepatitis B virus (HBs Ag) in human serum or plasma.
- INNOTEST® HCV Ab IV: Innogenetics Ltd, Gent, Belgium. An enzyme immunoassay for the detection of antibodies to human hepatitis C virus (HCV) in human serum or plasma.
- Antinuclear Antibody (ANA) Screen: DRG Diagnostics, Germany. Enzyme immunoassay for the qualitative screening on anti-ANA-antibodies in human serum or plasma.

Eligible participants were males and females 18 to 60 years of age (18–50 years for Protocol V001) who passed the assessment of understanding test and were willing and able to provide written informed consent. They did not engage in behavior that was high-risk for HIV infection; and were willing to undergo HIV counseling and testing, and to receive HIV test results. They also had to be willing to comply with the study procedures. Females were not to be pregnant or breastfeeding and had to be willing to prevent pregnancy during their participation in the trial.

High-risk behaviors for HIV infection within the previous 6 months as defined below were exclusionary:

- had unprotected vaginal or anal sex with a known HIV-infected person or a casual partner (ie, no continuing established relationship);
- engaged in sex work for money or drugs;
- used illicit drugs;
- acquired a sexually-transmitted disease (eg, *Gonorrhea, Chlamydia*, syphilis, *Trichomonas vaginalis*, or symptomatic *Herpes genitalis*);
- had a high-risk partner at the time of screening or within the previous 6 months (Protocol V001 only).

With regard to laboratory parameters, the participants were required to be negative for HIV Ab, hepatitis C Ab, syphilis serology, hepatitis B surface antigen, ANA; and for females, urine pregnancy test. Eligible participants were required to have normal hematology, biochemistry, CD4, and CD8 cell counts according to designated laboratory reference ranges, as well as normal urinalysis. For Protocol 010, mild/grade 1 abnormalities found in hematology, biochemistry, and urine parameters that were judged as not clinically significant by the principal investigator were allowed. In Protocol V001, mild/grade 1 elevations of ALT (SGPT), ie, elevated >1.25× upper limit of normal (ULN), mildly reduced neutrophil counts (up to 1001 cells/mm^3^), mildly reduced platelet counts (up to 125 cells/mm^3^), and mildly reduced hemoglobin values (11 g/dL) in females were permitted. Eosinophil counts, serum AST (SGOT), alkaline phosphatase, and total and direct bilirubin assays were eliminated from the screening tests for Protocol V001. Protocol V001 required that at least 30% of the enrolled volunteers be female.

Acute and minor abnormalities detected during the screening, such as upper respiratory tract infections and urinary tract infections were medically managed at the research centre. Conditions requiring follow-up, such as a cardiac murmur or hepatitis B infection, were referred to a nearby national referral hospital, the Kenyatta National Hospital, in Nairobi.

Clinic and laboratory personnel who were trained in GCP and GCLP, respectively, conducted all clinical trials according to ICH/GCP guidelines and the site's standard operating procedures.

## Results

For comparative purposes, Table 1 provides the consensus intervals for chemistry and hematology parameters for healthy adults in East and South Africa [8], the US-based comparison intervals used in that study [7], along with comparison intervals used for Protocols 010 and V001. Only a subset of all laboratory parameters is included.

**Figure 1:**
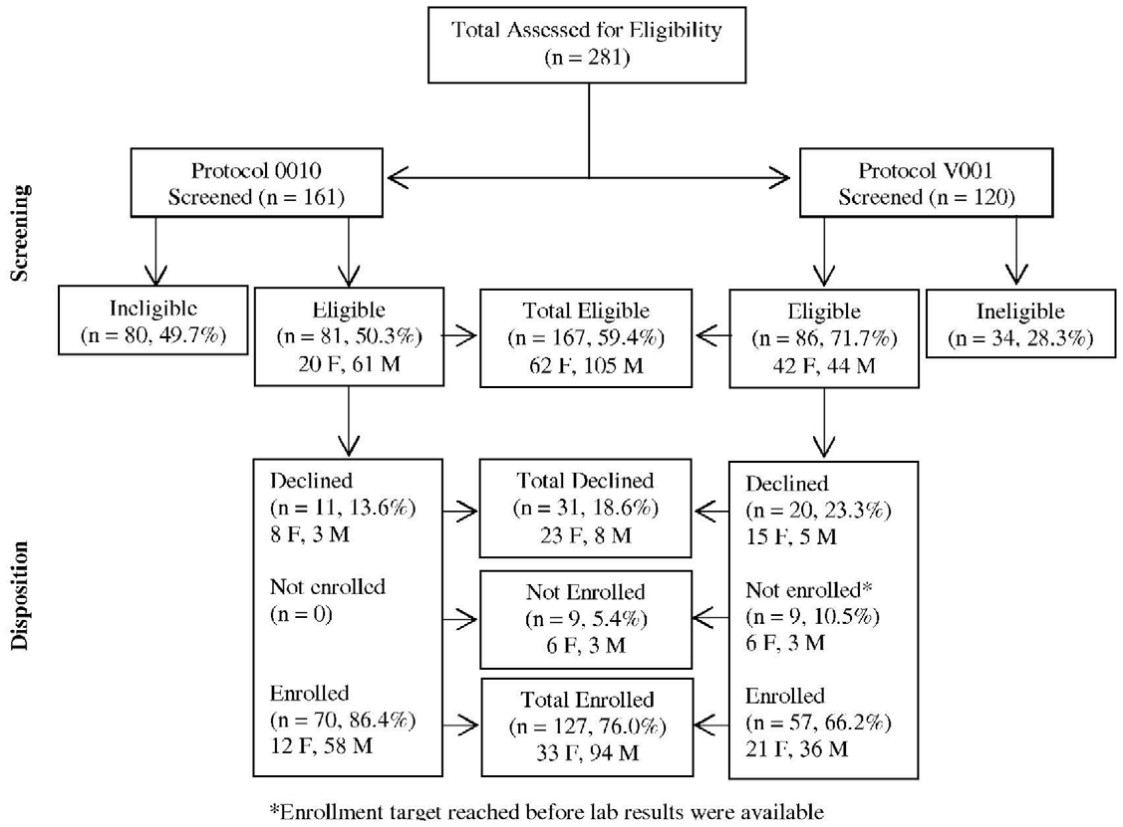
Disposition, by gender, of participants screened in two HIV vaccine clinical trials conducted in Nairobi.

Two hundred eighty-one participants were screened between the years 2003 and 2006 for the two referenced clinical trials (Figure 1). Of these, 167 (59.4%) were eligible for participation based on inclusion and exclusion criteria; there were 105 (62.9%) males and 62 (37.1%) females. Of those eligible, 127 (76.0%) were enrolled: 94 (74.0%) males and 33 (26.0%) females. Thirty-one (18.6%) of the eligible volunteers declined enrolment in the clinical trials. The majority of these 23/31 (74.2%) were females and most were from Protocol V001 (Figure 1). The recruitment rate averaged 13 volunteers per month for Protocol 010 and 14 for Protocol V001.

Those who were excluded (61.4%) because of abnormal biochemical or hematology values had no other diagnosed clinical condition (Table 2). Medical abnormalities and other conditions accounted for the remaining 38.6% of exclusions.

**Table 2:** Reasons for ineligibility in two HIV vaccine clinical trials in Nairobi.

	Number Ineligible by Protocol (n)		
	010	V001	Total
Reasons for Ineligibility	(80)	(34)	(114)
**Medical abnormality** – n (%)	20 (25.0)	15 (44.1)	35 (30.7)
Clinical history/PE	12 (15)	3 (8.8)	15 (13.2)
HIV Positive – n (%)	2 (2.5)	7 (20.6)	9 (7.9)
Hep B/C positive – n (%)	2 (2.5)	5 (14.7)	7 (6.1)
ANA – n (%)	4 (5.0)	NA	4 (3.5)
**Laboratory abnormality** – n (%)	55 (68.8)	15 (44.1)	70 (61.4)
Hematology – n (%)	23(28.8)	13 (38.2)	36 (31.6)
Biochemistry – n (%)	32(40.0)	2 (5.9)	34 (29.8)
**Other reasons** n – n (%)	5 (6.3)	4 (11.8)	9* (7.9)

*1 pregnancy, 5 high-risk behavior, 3 unable to comply with visit schedule.

PE =  physical examination.

Medical abnormalities alone accounted for 35 (30.7%) of all the reasons for ineligibility. Fifteen were excluded because of medical history and physical examination findings including allergy, bronchial asthma, and cardiac conditions. Serologic tests led to exclusion of an additional 20 (17.5%) participants: 9 HIV, 7 hepatitis B/C, and 4 ANA. In Protocol V001, 14.7% and 20.6% of exclusions were due to hepatitis B or C and HIV infections, respectively (Table 2). Other reasons for ineligibility in both protocols accounted for 7.9% of the exclusions and included reports of high-risk behavior for HIV infection (5), inability to comply with study visit schedules (3), and pregnancy (1).

Thirty-six (31.6%) of the total exclusions in the two studies were because of abnormal hematology values alone, with neutropenia accounting for the majority of these. Abnormal biochemistry values alone accounted for 34 of the 114 (29.8%) total exclusions (Table 2). Of 55 participants screened out through laboratory testing for Protocol 010, 28 (50.9%) had hyperbilirubinemia (Table 3).

**Table 3:** Ineligibility due to abnormal hematology and biochemistry values in two HIV vaccine clinical trials.

	010	V001	Total
Parameter – n (%)	n = 55	n = 15	n = 70
**Hematology**			
Low hemoglobin	1 (1.8)	4 (26.7)	5 (7.1)
Leucopenia	1 (1.8)	0	1 (1.4)
Neutropenia	11*(20.0)	9 (60.0)	20 (28.6)
Eosinophilia	6 (10.9)	NA	6 (8.6)
Low CD4 counts	4 (7.3)	NA	4 (5.7)
**Total Hematology**	23 (41.8)	13 (86.7)	36 (51.4)
**Biochemistry**			
Elevated total bilirubin	28+(50.9)	NA	28 (40)
Elevated ALT	1 (1.8)	2**(13.3)	3 (4.3)
Alkaline phosphatase	3 (5.5)	NA	3 (4.3)
**Total Biochemistry**	32 (58.2)	2 (13.3)	34 (48.6)

*Two cases had concurrent leucopenia.

+Five cases were due to high total serum bilirubin and neutropenia; 4 had elevated alkaline phosphatase.

**ALT elevated >1.25×ULN.

Nineteen of the 28 participants with abnormal total bilirubin values and 11 of 20 with neutropenia would have been eligible had the East/South Africa consensus intervals been used (Table 4). All of these participants were from Protocol 010.

**Table 4:** Participants with selected laboratory abnormalities: those who would have qualified using East/South Africa consensus intervals.

	East/South Africa Consensus Interval *	Modified US Comparison Interval	Number Ineligible per US Comparison Interval	Number Ineligible per East/South Africa Consensus Interval	Number projected eligible per East/South Africa Interval
Total Bilirubin (µmol/L)	2.9–37.0	<17	28	9	19+
Neutropenia (×10^3^ cells/µL)	1.0–5.3	1.5–8.0	20	9	11
Total WBC (×10^3^ cells/µL)	3.1–9.1	3.3–11.0	1	0	1
Hemoglobin: female (g/dL)	9.5–15.8	12–18	5	4	1
Total			54	22	32

*From Karita, et al, [8].

+ Total bilirubin was >17 and <25.5 µmol/L for 12 participants and >25.5 and <37 µmol/L for 7 participants.

## Discussion

Participant recruitment for HIV clinical trials is a difficult and rigorous process that is very demanding of staff time and financial resources. The participant recruitment rates for Protocols 010 and V001 were approximately three times faster than the studies completed in 2001 and 2002 [6]. This was likely due to lessons learned with recruitment.

The main reasons for ineligibility during volunteer screening for the clinical trials included abnormal laboratory parameters and medical abnormalities. Abnormal hematology and biochemistry values contributed the most (61.4%) to screen failures in otherwise clinically healthy participants. The high rate of exclusions based on laboratory parameters raises some questions: Could there have been un-diagnosed co-morbidities? Were the laboratory reference ranges unrealistic for this population?

Not all possible endemic co-morbidities were included when screening, such as helminthiasis and malaria. It is possible that some of these may have contributed to some of the laboratory abnormalities noted. For instance, in one study among adult African volunteers from East and Southern Africa, possible malaria accounted for 6% of exclusions [9].

It is worth noting that most of the abnormalities in total bilirubin and neutrophil counts were not in combination with other abnormal laboratory parameters as would be expected if the causes were undiagnosed clinical conditions. Of the 28 participants with abnormally increased total bilirubin results, there were only 4 with abnormal liver function test values (increased alkaline phosphatase) hence for the majority, reduced uptake of bilirubin due to liver pathology is an unlikely explanation. Hemolysis is also an unlikely explanation because none of the participants with elevated total serum bilirubin was clinically jaundiced or had abnormal red cell indices.

The notable difference in the contribution of hematology and biochemistry to the overall laboratory abnormalities between Protocol 010 (68.8%) and Protocol V001 (44.1%) as seen in Table 2 is mostly due to the removal of total serum bilirubin, alkaline phosphatase, CD4 cell count, and eosinophil assays from the screening laboratory parameters for the Protocol V001. Having realized from earlier studies that elevated total serum bilirubin or eosinophilia in many individuals from this population were not clinically significant, these assays were not included in the screening tests. Yet, the average monthly recruitment rate of participants in the two protocols remained similar and was better than that of earlier studies in which Omosa, et al[6], reported recruitment rates of approximately 4 to 5 participants per month. Further studies to determine reasons for the improvement in the recruitment speed need to be carried out to better plan for future recruitment strategies, especially for the larger Phase 3 trials.

Because of poor access and the expense, people in developing countries do not routinely seek medical attention unless signs and/or symptoms of disease appear. The presence of asymptomatic co-morbidities, such as intestinal helminthiasis, in such a population cannot be ruled out; however, it would not be practical to routinely screen for all potential co-morbidities during the conduct of a clinical trial, especially during phase III trials involving large numbers. Furthermore, when the next candidate HIV vaccine becomes available for testing, potential participants should not be excluded because of such minor variations. In such a situation, a clinically ‘normal’ population would include those with suspected/assumed asymptomatic co-morbidities. Studies have also shown that laboratory parameters vary geographically; reasons include diet, altitude, race, gender, and age [8], [10], [11], [12], [13], [14], [15]. Therefore, using laboratory reference ranges derived from populations in industrialized countries to assess participant eligibility and safety for clinical trials in an African population could lead to unnecessary exclusion of otherwise eligible participants and contribute to over-reporting of adverse events. Had the East/South Africa consensus intervals [8], [9] been used for reference, 32 of previously ineligible participants would have qualified for these two studies (Table 4); this would have lead to an overall exclusion rate due to abnormal laboratory parameters of 46.3% (38/82) in contrast to 61.4% (Table 2). At the time these studies were conducted, Karita, et al [8], had not yet reported their data establishing consensus ranges for adults in East and South Africa.

To lose otherwise qualified participants because of laboratory values that are considered abnormal by reference standards from another part of the world has significant impact on resources and requires further consideration. Thus, it is essential that laboratory reference ranges generated from local populations be used to conduct clinical trials, as well as to manage patient care.

Although the participants screened were considered to be low risk for HIV and other sexually transmitted infections (STI), the rates of hepatitis B or C and HIV infections were relatively high. Such findings call for review of the HIV/STI risk assessment tool employed in selecting low-risk participants for such trials. Worth noting, there was only one pregnancy at screening in this population, likely due to self-selection of subjects after pre-trial counseling and education sessions when potential participants became fully aware of the study requirements. Interestingly, there seems to have been less self-exclusion of HIV sero-positive participants, which points to a general lack of knowledge regarding self-HIV status in this population at the time. It is hoped that with the ongoing HIV voluntary counseling and testing, and provider-initiated counseling and testing campaigns in Kenya, self-knowledge of HIV sero-status has since improved.

Other challenges pertaining to participant recruitment became evident: the high numbers of eligible participants declining enrolment and the low female: male ratio (Figure 1). Some of the declines were due to negative influences from peers; most were females. There was a low female: male ratio in the Protocol 010, in which there was no minimum requirement for female representation (Figure 1). The requirement that female participants should not be pregnant or breastfeeding during participation may have partly contributed to this; other cultural and practical influences may also have impacted female participation. These observations need further study.

In addition, some potential participants were greatly disappointed when they learned they did not qualify for enrolment in the trials. Individuals who appear eager to participate are more likely to continue and complete a trial. Using reference ranges established from local populations would avoid unnecessary exclusion of some of these otherwise willing participants.
